# Angularly quantized spin rotations in hexagonal LuMnO_3_

**DOI:** 10.1038/s41598-022-06394-2

**Published:** 2022-02-14

**Authors:** Seung Kim, Jiyeon Nam, Xianghan Xu, Sang-Wook Cheong, In-Sang Yang

**Affiliations:** 1 Department of Physics, Ewha Womans University, Seoul, Korea; 2 Rutgers Center for Emergent Materials and Department of Physics and Astronomy, Rutgers University, Piscataway, NJ USA

**Keywords:** Nanoscience and technology, Physics

## Abstract

Optical control of the spin degree of freedom is often desired in application of the spin technology. Here we report spin-rotational excitations observed through inelastic light scattering of the hexagonal LuMnO_3_ in the antiferromagnetically (AFM) ordered state. We propose a model based on the spin–spin interaction Hamiltonian associated with the spin rotation of the Mn ions, and find that the spin rotations are angularly quantized by 60°, 120°, and 180°. Angular quantization is considered to be a consequence of the symmetry of the triangular lattice of the Mn-ion plane in the hexagonal LuMnO_3_. These angularly-quantized spin excitations may be pictured as isolated flat bubbles in the sea of the ground state, which may lead to high-density information storage if applied to spin devices. Optically pumped and detected spin-excitation bubbles would bring about the advanced technology of optical control of the spin degree of freedom in multiferroic materials.

## Introduction

Application of spin control is looking forward to expanding its horizon to unprecedented level of data processing and storage capability^1,2^. Understanding the spin–spin interaction in magnetic materials are important and required in order to control the spin degree of freedom in the materials. However, most investigations trying to discover the origin of magnetic properties have been encountered with mixed information of unknown origin, which make it hard to analyze the genuine response from the magnetic system under investigation.

Despite the aforementioned difficulty, magnetic technologies have been actively explored in various fields to overcome the practical issues with the *R*MnO_3_ (*R* = rare earths, Y, and Sc) which are well known materials possessing ferroelectric and antiferromagnetic (AFM) transitions simultaneously. This binary character has been investigated by several optical and magnetic techniques as well as theoretical studies because of its immense potential for application^3–7^. Most *R*MnO_3_ materials exhibit a new possibility of tuning the response of the electric parameters to a magnetic field and vice versa, due to strong coupling of electric and magnetic order parameters^8–13^.

The crystal structure of hexagonal *R*MnO_3_ in P6_3_*cm* space group leads to split the 3*d* energy levels of Mn^3+^-ions, due to change in the crystal field symmetry below the Curie–Wiess temperature T_C_ (> 700 K)^14–17^. A Mn^3+^-ion has four electrons in 3*d* orbital (total S = 2). When a proper energy is supplied to the system, one of the electrons in ${d}_{{x}^{2}-{y}^{2}}$/${d}_{xy}$ levels can be excited to the ${d}_{{3z}^{2}-{r}^{2}}$ level, called Mn *d*–*d* transition^11,16–18^.

The Mn^3+^-ions are placed at $\mathrm{x}=\frac{1}{3}$ position of the triangular lattice in a *xy* plane in paramagnetic phase. However, below Néel temperature T_N_ (< 100 K), they move out of the ideal site due to Mn trimerization^15,19–21^, giving rise to two different spin–spin interaction integrals, intra-trimer interaction *J*_1_ and inter-trimer interaction *J*_2_.

Raman spectroscopy is based on the inelastic scattering related to electronic transitions in a material. Raman selection rule can help establish the excitations of the magnetic origin with high resolution^22,23^. Previous Raman studies show that the spin excitations strongly correlated with a particular electronic transition are observed in hexagonal *R*MnO_3_ below the T_N_^24–28^. Spin excitations of relatively high energy (~ 0.1 eV) are optically excited in the hexagonal *R*MnO_3_ system through the resonance with the Mn *d*–*d* transition^24,28–30^. These are not phonons at the zone boundaries that could fold in due to structural or magnetic ordering.

LuMnO_3_ is of the popular hexagonal manganite family crystalized in the P6_3_*cm* group below T_N_^6,14,20,31^. Unlike other rare-earth hexagonal *R*MnO_3_, only Mn^3+^-ion has spins in hexagonal LuMnO_3_^10,32^, and the spin excitations in the Mn-ions can be well separated without screening or overlapped effects by other than Mn-ion spins. Diverse articles have been reported regarding this material for the latest several years, focusing on the ensemble of the lattice constant, crystal structure, spin structure, phonon, magnon and spin exchange integrals^9,12,13,15^.

In this study, we present a microscopic explanation for the origin of spin excitations based on a simple spin-spin interaction Hamiltonian in hexagonal LuMnO_3_ system. We propose a model associated with the spin rotation of the Mn ions in the symmetry of the triangular lattice with the AFM ordering. Our model can explain the spin excitation peaks observed in LuMnO_3_ within the framework of the spin-spin interaction in the Mn-trimer network below T_N_.

## Results

**Figure 1:**
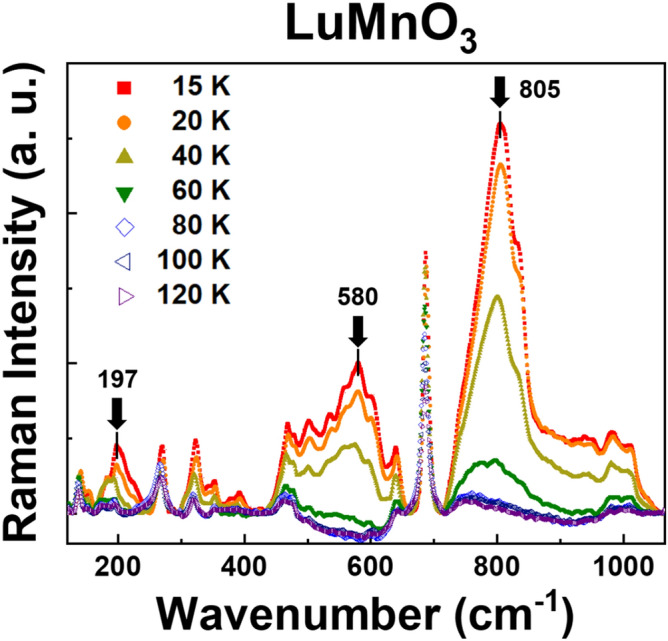
Temperature dependence of Raman spectra of hexagonal LuMnO_3_ single crystal in the cross scattering configuration. Several broad peaks (~ 197, 580, 805 cm^−1^) are prominent at low temperatures, and disappear above 80 K (~ T_N_).

Figure 1 shows temperature-dependent Raman spectra of the hexagonal LuMnO_3_ single crystal in cross polarization scattering geometry from 120 to 1050 cm^−1^ range as a function of temperature. All the spectra were normalized by the intensity of the A_1_ phonon at ~ 680 cm^−1^. The A_1_ phonon should be forbidden in the cross polarization, except in the resonance condition. Other A_1_ (~ 266, 475 cm^−1^), E_1_ (~ 640 cm^−1^), and E_2_ (~ 318, 350, 461 cm^−1^) phonon modes observed in the spectra are consistent with previous results for LuMnO_3_^12^. Besides these phonons, several broad peaks (~ 197, 580, 805 cm^−1^) are prominent at low temperatures. These broad peaks disappear above a critical temperature ~ 80 K. (See the Supplementary Information.) As in various hexagonal *R*MnO_3_, AFM spin ordering appears under ~ 100 K, and the T_N_ values for LuMnO_3_ suggested by previous papers are consistent with the critical temperature ~ 80 K^24,33,34^. This strongly suggests that these broad peaks are in harmony with the AFM spin ordering in LuMnO_3_. In the sense that the peaks in the Raman scattering spectra measure the energy difference between the ground state and the excited state, it is reasonable to assume that the broad peaks represent the energy difference between the ground state and several excited states of the AFM spin ordering. The peak at 805 cm^−1^ may be considered as the spin-flip excitation energy for the Mn^3+^ ions in one triangle of the lattice (trimer) in LuMnO_3_ just as the 760 cm^−1^ peak in HoMnO_3_ is claimed to be due to the spin-flip excitation^28^. What about the origin of the other broad peaks at lower wavenumbers? In order to answer the question, we first need to look for the physical parameters from the relationship between the measured Raman peak at 805 cm^−1^ and the Hamiltonian for the spin-flip excitation of the Mn^3+^ ions in the trimer.

The spin excitation peaks in hexagonal *R*MnO_3_ have been observed by red lasers only^24,29,30^. The optical conductivity measurements on hexagonal *R*MnO_3_ provide convincing evidence that the Mn *d*–*d* transition take place 1.5–1.8 eV at room temperature^11,16–18^. In LuMnO_3_, Mn *d*–*d* transition would occur around 1.615 eV at 300 K, and the Mn *d*–*d* transition peak blue shifts about 0.15 eV at 10 K^16^. Red excitation lasers of 620–700 nm wavelength supply the energies corresponding to 1.77–2 eV, close to the energies of Mn *d*–*d* transition of LuMnO_3_ at 10 K. Many Raman scattering studies on hexagonal *R*MnO_3_ system support our interpretation of resonance with Mn *d*–*d* transition well^24,29,30^. The resonance Raman scattering is specific only to the Mn *d–d* transition, so that the spin excitations observed by the red lasers are independent of the disturbance from the excitations of the *R* ions. Therefore, the resonance Raman scattering would open a new opportunity to study the magnetic properties associated with the Mn ions selectively in hexagonal *R*MnO_3_ system.

The resonance effect with the Mn *d*–*d* transition further supports that these broad peaks observed in LuMnO_3_ below T_N_ may be from the excitation of the Mn-ions. But are they due to the excitation in the spin structure of Mn-ions? Let us consider possible excited Mn^3+^-ion spin configurations in triangular lattice within the AFM ordering symmetry. Our aim is to calculate the excitation energy $\Delta \mathrm{E}$, and match with the energies of the broad peaks observed in the Raman spectra.

**Figure 2:**
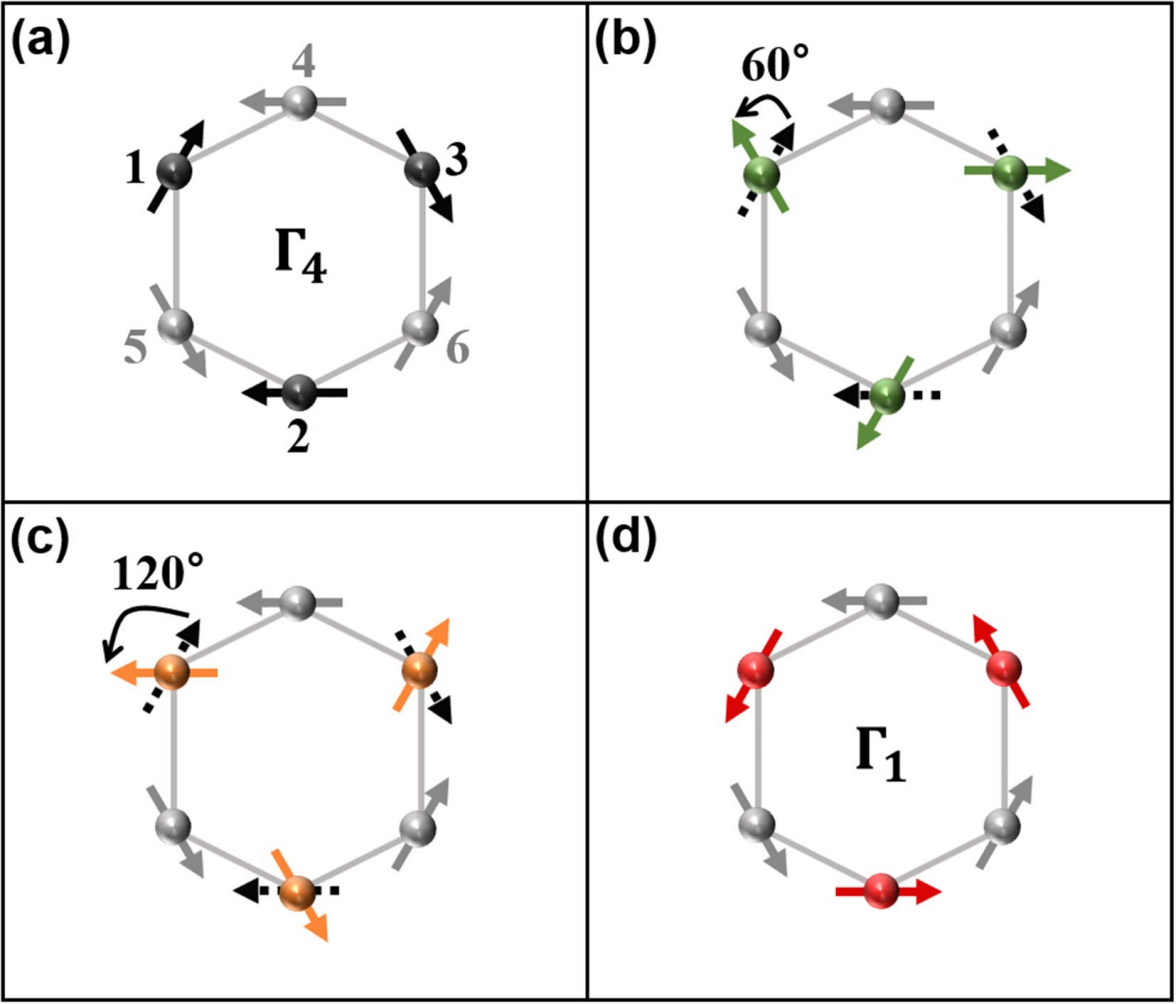
Several AFM spin configurations of hexagonal LuMnO_3_ single crystal. Mn-ions are indicated by solid spheres: gray spheres (number 4, 5, 6) are located in the $z=\frac{c}{2}$ plane and black or colored ones (number 1, 2, 3) are in the $z=0$ plane. Each arrow overlapped with the sphere expresses the spin direction of a Mn^3+^-ion (S = 2). We assume that colored spheres only are excited by the incident light. (**a**) The ${\Gamma }_{4}$ spin structure in the ground state. (**b**) Three spins (1, 2, 3) rotated counterclockwise by 60° (green arrows), or (**c**) by 120° (orange arrows). (**d**) Three spins (1, 2, 3) are flipped from the ${\Gamma }_{4}$ structure, resulting in the ${\Gamma }_{1}$ spin structure locally (red arrows).

Mn *d*–*d* transition would induce a transient excited state of different spin symmetry. The new transient spin-ordered state should be consistent with the AFM structure of the P6_3_*cm* space group. Spin structures in this manuscript are described by unidimensional $\Gamma$ representations as in diverse literatures^19,21,31^. Among the representations, ${\Gamma }_{4}$ is the most plausible candidate for the ground state of LuMnO_3_ as displayed in Fig. 2a^20,34,35^. The Mn-ion spins are arranged on a trimer with counterclockwise configuration in the $\mathrm{z}=\mathrm{c}/2$ plane (gray solid spheres), while those are arranged with clockwise configuration in the $\mathrm{z}=0$ plane (black solid spheres). Layered MnO_5_ bipyramids are separated far enough to ignore the interaction of the Mn-ions between the planes, so the main concern would be interactions in one *xy* plane^32,35^. In this manuscript, we will assume that three Mn ions of a trimer in the $\mathrm{z}=0$ plane only are excited by the resonant light.

Raman selection rule is resulted from the conservation of total angular momentum, $\overrightarrow{J}= \overrightarrow{L}+\overrightarrow{S}$. Here, $\overrightarrow{J}$ is total angular momentum, $\overrightarrow{L}$ is orbital angular momentum, and $\overrightarrow{S}$ is spin angular momentum. Raman scattering has a two-photon process which should satisfy $\Delta \mathrm{J}=0\,\mathrm{ or }\,\pm 2$. As was explained above, resonance Raman scattering in hexagonal *R*MnO_3_ is linked with Mn *d*–*d* transition, so $\Delta \mathrm{L}$ would remain unchanged, which requires $\Delta \mathrm{S}=0\,\mathrm{ or }\,\pm 2$. However, the probability of approaching $\Delta \mathrm{S}=\pm 2$ while maintaining the AFM spin ordering would be low, due to the frustration condition imposed by the triangular lattice. As a result, $\Delta \mathrm{S}=0$ is the most probable transition allowed within the AFM ordering as well as Raman selection rule. It would be possible to satisfy both conditions if all the three spins in one trimer rotate simultaneously by the same angle in the same direction. The symmetry of the triangular lattice permits only certain angles of rotation of the spins, namely, 60°, 120°, and 180°. These three rotations are illustrated in Fig. 2b–d. Details of argument on the angular quantization of the spin-rotational excitation are given in the Supplementary Information. Number 1, 2, 3 spins are rotated counterclockwise from the spin structure of ${\Gamma }_{4}$ symmetry shown in Fig. 2a, by 60° (Fig. 2b, green arrows), by 120° (Fig. 2c, orange arrows), and by 180° (Fig. 2d, red arrows), after excitation. Especially if all the three spins in one trimer are rotated 180° (Fig. 2d), which is identical with the flipping of all three spins, the symmetry of the spin ordering would change from ${\Gamma }_{4}$ to ${\Gamma }_{1}$ representation locally. In analogy, these may be regarded as the ${\Gamma }_{1}$ bubbles in the ${\Gamma }_{4}$ sea.

**Figure 3:**
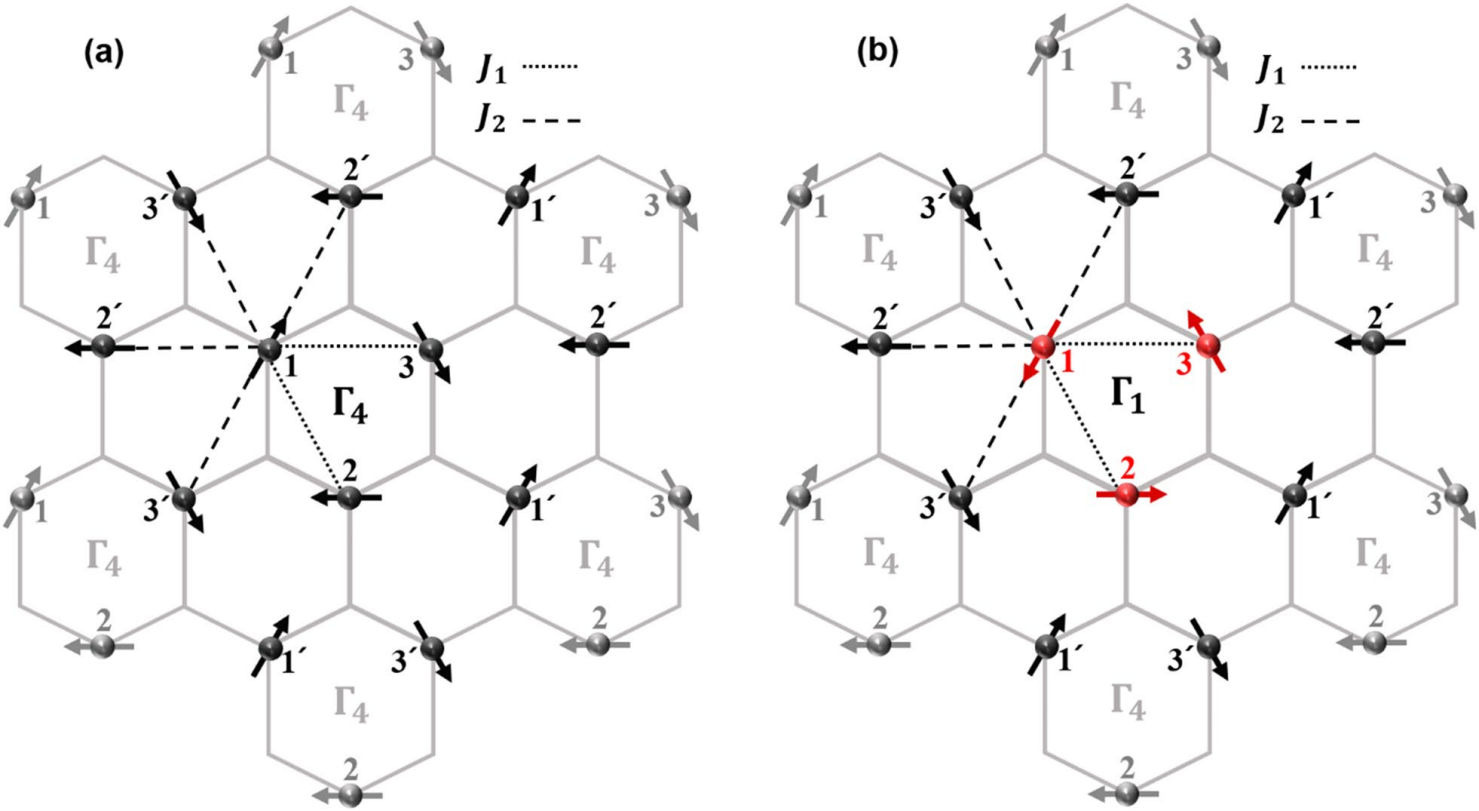
A trimer composed of three Mn-ions (center), and its six neighbors in the $z=0$ plane in hexagonal LuMnO_3_ single crystal. Below T_N_, each trimer has two kinds of spin exchange integral: Dotted lines (nearest neighbor) indicate spin exchange integral ${J}_{1}$, and dashed lines (next nearest neighbor) indicate spin exchange integral ${J}_{2}$. (**a**) Every trimer has ${\Gamma }_{4}$ spin structure in the ground state. (**b**) Norm trimer has the ${\Gamma }_{1}$ spin structure locally after the spin-flip excitation, while its six neighbors remain in the ${\Gamma }_{4}$ spin structure.

A simple Hamiltonian is suggested below to address the spin excitations involving rotation of the spins in hexagonal LuMnO_3_ system. The Hamiltonian includes two terms for the spin interactions; first term is the spin–spin interaction between the Mn-ions within a trimer with spin exchange integral ${J}_{1}$ (intra-triangular interaction), and second is that between the Mn-ions in neighboring triangles with ${J}_{2}$ (inter-triangular interaction). Figure 3 shows a concept of the model (Fig. 3a) in the ground state (${\Gamma }_{4}$) and that (Fig. 3b) in one of the excited states (${\Gamma }_{1}$). There are six other trimers (${\Gamma }_{4}$) around a norm trimer. Below T_N_, Mn trimerization contracting in the Mn-ion *xy* plane is found in LuMnO_3_, which makes it possible to distinguish ${J}_{1}$ and ${J}_{2}$^15,19–21^. In Fig. 3a, number 1 spin has two nearest neighbors (number 2 and 3) connected with dotted line (intra-triangular interaction ${J}_{1}$) and four next nearest neighbors (number 2′ and number 3′) connected with dashed line (inter-triangular interaction ${J}_{2}$). Likewise, each of number 2 spin and number 3 spin has also two ${J}_{1}$ and four ${J}_{2}$ interactions. Considering double counting, the Hamiltonian should be as follows;

1 $${\text{H}} = { }J_{1} \left( {\mathop \sum \limits_{{\begin{array}{*{20}c} {i,j = 1} \\ {\left( {i \ne j} \right)} \\ \end{array} }}^{3} \vec{S}_{i} \cdot \vec{S}_{j} } \right) + { }2J_{2} \left( {\mathop \sum \limits_{{\begin{array}{*{20}c} {i, j = 1} \\ {\left( {i \ne j} \right)} \\ \end{array} }}^{3} \vec{S}_{i} \cdot \vec{S}_{j^{\prime}} } \right)$$

where ${\overrightarrow{S}}_{i}$ is the Mn^3+^-ion spin in one trimer, and $\vec{S}_{j\prime }$ is that in six neighboring trimers.

The largest energy difference from ground state is derived from the Hamiltonian when the number 1, 2, 3 spins are rotated 180 degrees, namely, three-spin flipping. Additionally, other notable energy values are corresponding to rotation of the spins by 60° and 120°, and they are listed in Table 1. First, it suggests energy differences, $\Delta \mathrm{E}$, calculated by the Eq. (1). When the three spins in a trimer are rotated by the same angle simultaneously, there is no cost in the energy related to the stronger spin–spin interaction ${J}_{1}$. Thus $\Delta \mathrm{E}$ is determined by the weaker interaction ${J}_{2}$ only. Calculation based on the model is performed taking total S = 2 of a Mn^3+^-ion and assuming the excitation energy of the three-spin flipping is corresponding to the broad peak at ~ 805 cm^−1^ in Fig. 1^28^. From our model, we could obtain the value of ${J}_{2}=2.08$ meV, which is in reasonable agreement with various researches dealing with hexagonal LuMnO_3_^20,33^. It is notable that the excitation energies due to three-spin rotation by 60° and 120° precisely describe the broad peaks at 197 cm^−1^ and 580 cm^−1^ in Fig. 1, respectively.

**Table 1:** Excitation energy values calculated from the model Hamiltonian in the text corresponding to the rotation angles of the three Mn-spins in a trimer in LuMnO_3_ single crystal.

Rotation angle	$\Delta \mathrm{E}$	Model calculation		Experimental data	
		(meV)	(cm^−1^)	(meV)	(cm^−1^)
$60^\circ$	$3{S}^{2}{J}_{2}$	24.95	201.3	24.43	197
$120^\circ$	$9{S}^{2}{J}_{2}$	74.85	603.7	71.91	580
$180^\circ$	$12{S}^{2}{J}_{2}$	99.80	804.9	99.81	805

In the model, $S=2$ and ${J}_{2}=2.08$ meV (Park, J. et al., Oh, J. et al.) are assumed.

Supplementary Figs. 2a, c, e, and g clearly show that spin rotations of a Mn-ion trimer by 0°, 60°, 120°, and 180° preserve the triangular symmetry by sustaining 30° or 60° angles between the spin directions. On the other hand, spin rotations by other angles, for example, 30°, 90°, and 150° do not keep the triangular symmetry (Supplementary Figs. 2b, d, f). Consequently, only 60°, 120°, and 180° rotations of a trimer are allowed in the hexagonal crystal symmetry, and thus the spin excitation energies are quantized by the inherent triangular symmetry of the hexagonal LuMnO_3_^36^.

The spin excitation by 60°, 120°, and 180° rotation is a local excitation in one isolated trimer, not in the entire plane. These spin-rotational excitation of one Mn-ion trimer is like isolated flat bubble in the ground state of the ${\Gamma }_{4}$ sea. The spin symmetry of the bubble is locally different from the symmetry of the background.

**Figure 4:**
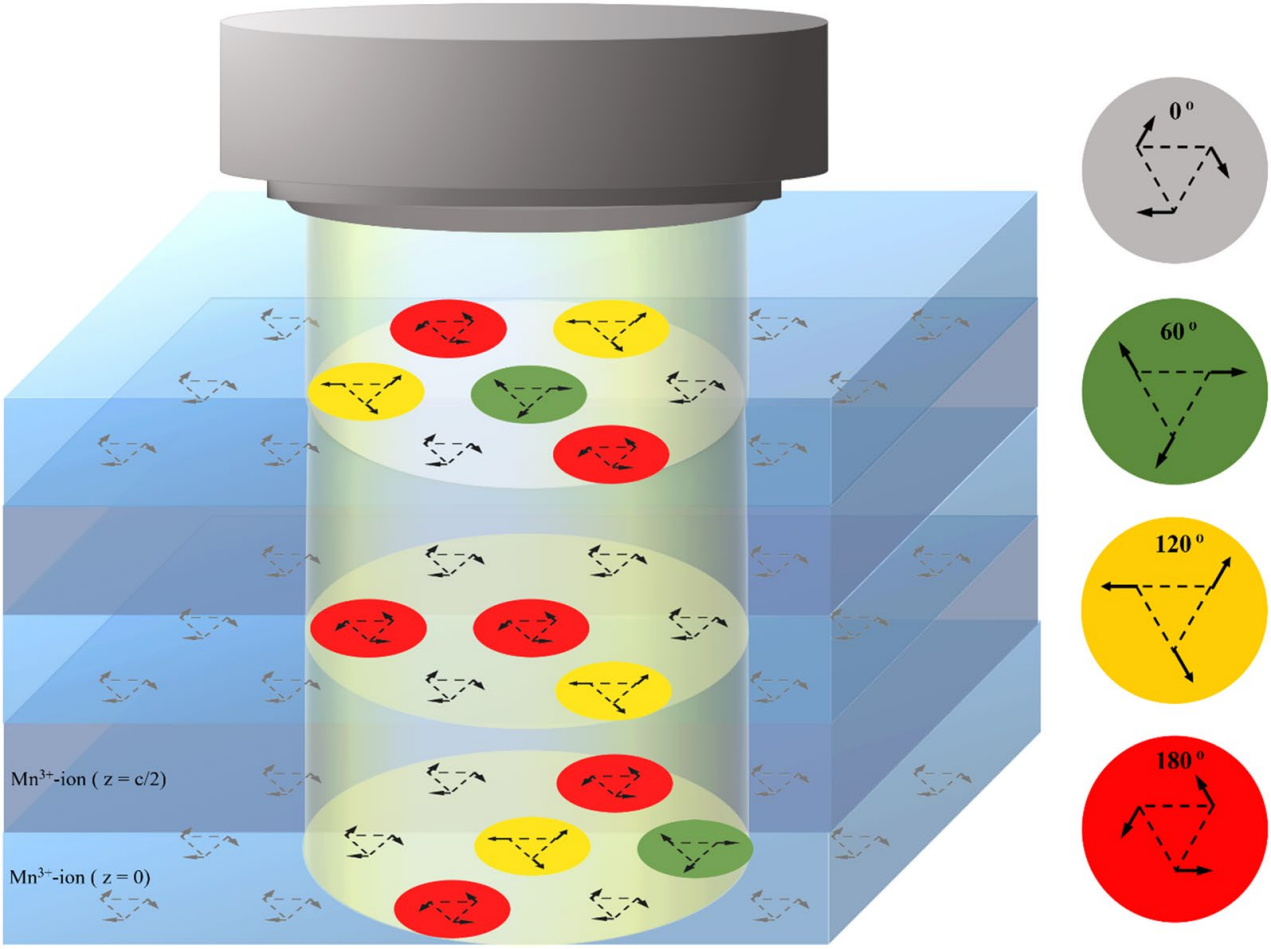
A visualization of the isolated spin-rotational excitations. Below T_N_, the Mn^3+^-ions are lying on each *xy* plane maintaining the ${\Gamma }_{4}$ spin structure. The spin states are excited by simultaneous rotation of the three spins in a trimer by 60° (green bubbles), 120° (orange bubbles), and by 180° (red bubbles) under the resonant light. The symmetry of the triangular lattice allows only these three rotations in hexagonal LuMnO_3_.

A three-dimensional cartoon of the collection spin-rotational excitation bubbles is presented in Fig. 4 to help understand the nature of the isolated spin excitations. The spin ground states are abundant enough to form the ${\Gamma }_{4}$ sea below T_N_. When a resonant light generating the Mn *d*–*d* transition is applied to a part of the ${\Gamma }_{4}$ sea, in-plane spin-rotational excitations would emerge with rotation angles of 60°, 120°, and 180°. These spin excitations are isolated from each other, and each isolated excitation could be considered as an isolated flat bubble in the ${\Gamma }_{4}$ sea as pictured in Fig. 4. 60° rotations are depicted as green bubbles, 120° and 180° rotations, orange and red bubbles, respectively. Angularly-quantized spin-rotational excitation constitutes an example of energy quantization by the symmetry allowance.

## Discussion

We suggest a model based on a spin–spin interaction Hamiltonian to explain the spin excitation peaks observed in the Raman spectra of hexagonal LuMnO_3_ in the cross configuration below T_N_. Broad Raman peaks of hexagonal LuMnO_3_ below T_N_ are excited through the resonance with the Mn *d*–*d* transition by the incident red laser (~ 1.85 eV). Our model for the spin excitation suggests simultaneous rotation of the spins of the in-plane Mn^3+^-ions to account for the energies of the Raman peaks. The model should meet several conditions: the Raman selection rule, preservation of the spin symmetry associated with the triangular lattice while maintaining the AFM spin ordering. A simple calculation is carried out to compare the model with our experimental Raman data. We could get a microscopic value for next nearest neighbor, ${J}_{2}=2.08$ meV, which is consistent with the results from the neutron scattering (${J}_{2}=1.54$ meV^33^) and theoretical calculations (${J}_{2}=2.37$ meV^20^, 3 meV^37^). Based on the ${J}_{2}$ values obtained, $\Delta \mathrm{E}$ values are calculated by the Hamiltonian Eq. (1), therefore, we could assign the broad peaks as the isolated spin excitations associated with the spin rotation by 60°, 120°, and 180°. The ground spin state and the three-spin-flipping state represent ${\Gamma }_{4}$ and ${\Gamma }_{1}$ configuration, respectively, with the energy difference of ~ 0.1 eV (corresponding to ~ 805 cm^−1^).

In this study, the spin excitation peaks of LuMnO_3_ observed in the Raman scattering are due to the excitations solely in the Mn spins through the resonance with the Mn *d*–*d* transition. Neutron scattering and magnetization measurements of *R*MnO_3_ (*R* = rare earths) on the other hand, are affected by the strong paramagnetic moment of the rare-earth ions, and the magnetic excitations by the Mn-ions are hard to differentiate from those related with the rare earths. That is, resonance Raman has a potential to differentiate the magnetic phase transition due to the Mn ions especially in hexagonal *R*MnO_3_ system with strong paramagnetic moment of rare earth *R*^3+^ ions other than Lu^3+^ ions. Raman spectroscopy resonant with Mn *d*–*d* transition suggests a good approach to study the spin ordering of the Mn ions in other hexagonal *R*MnO_3_.

All the spin excitation peaks observed in LuMnO_3_ below the Néel temperature by an inelastic light scattering are explained in terms of the Heisenberg spin–spin interaction Hamiltonian. We claim that the peaks at 197, 580, and 805 cm^−1^ are due to excitations by Mn-ion spins rotated by 60°, 120°, and 180°, respectively. The rotation angles are quantized by 60°, 120°, and 180°, which is a consequence of the symmetry of the triangular lattice. The spin excitations are isolated in each triangular lattice. The isolated spin excitations may lead to optical control of the spin degree of freedom in future.

## Materials and methods

Hexagonal LuMnO_3_ single crystal was grown using the traveling floating zone method and characterized by magnetization, resistivity, and x-ray powder diffraction^38^. Platelet sample was cleaved perpendicular to the c axis. The sample area was $2.0\ \mathrm{mm}\times 2.0\ \mathrm{mm}$ with $0.2\ \mathrm{mm}$ thickness. Helium-closed-cycle cryostat was used to control the temperature of the sample from 15 to 120 K in vacuum chamber. Raman scattering spectra were obtained by Horiba LabRam spectrometer coupled with a liquid-nitrogen-cooled CCD under $z(yx)\overline{z }$ cross configuration. Excitation light source was visible red laser which has continuous 671 nm (~ 1.85 eV) wavelength, with the power of 40 mW on the chamber window. Laser spot radius was about $0.8 \ \mathrm{mm}$ when using × 40 objective lens. The background is subtracted from the raw Raman spectra and Adjacent-Average smoothing is performed by window size 7, threshold 0.05 after the subtraction. Whole data are normalized by the A_1_ phonon (~ 680 cm^−1^) intensity and we considered temperature dependence of the A_1_ phonon^12^ for each temperature when normalizing the spectra.

## Supplementary Information

Supplementary Information.
