# Supplementary material for: Angularly quantized spin rotations in hexagonal LuMnO3
Source: Sci Rep. 2022 Feb 14;12:2424. doi: 10.1038/s41598-022-06394-2 (PMC8844039; doi:10.1038/s41598-022-06394-2)
Supplement: Supplementary file 1 — Supplementary Information. [file 41598_2022_6394_MOESM1_ESM.docx]

**Supplementary Information**

**Angularly Quantized Spin Rotations in Hexagonal LuMnO_3_**

Seung Kim^1^, Jiyeon Nam^1^, **Xianghan Xu**^2^, Sang-Wook Cheong^2^, and In-Sang Yang^1,*^

*^1^Department of Physics, Ewha Womans University, Seoul Korea
^2^Rutgers Center for Emergent Materials and Department of Physics and Astronomy, Rutgers University, New Jersey, USA*

* Corresponding Author: [yang@ewha.ac.kr](mailto:yang@ewha.ac.kr)


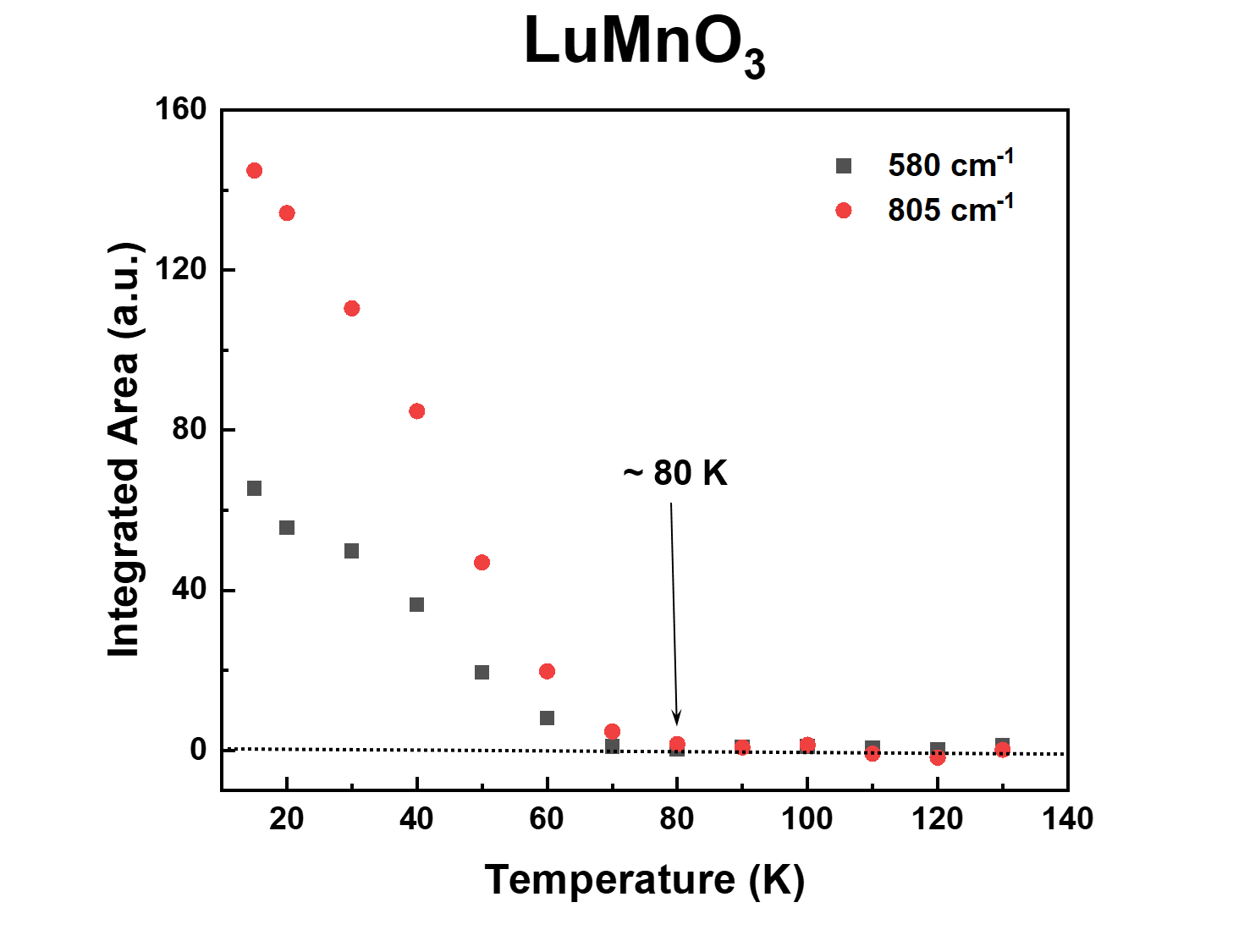


Supplementary Figure 1. Identification of the T_N_ of hexagonal LuMnO_3_ single crystal deduced from the temperature dependence of the spin excitation peaks at 580 cm^-1^ (filled squares) and 805 cm^-1^ (filled circles) in the Raman spectra.

Integrated intensities of spin excitation peaks (~ 580, 805 cm^-1^) at different temperatures are shown in the Supplementary Figure 1. The intensities are zero at high temperatures and they start to deviate from zero below a certain temperature, which we identify as the spin-ordering temperature T_N_. Deviation from the zero line (dotted horizontal line) indicates that T_N_ would be close to 80 K.


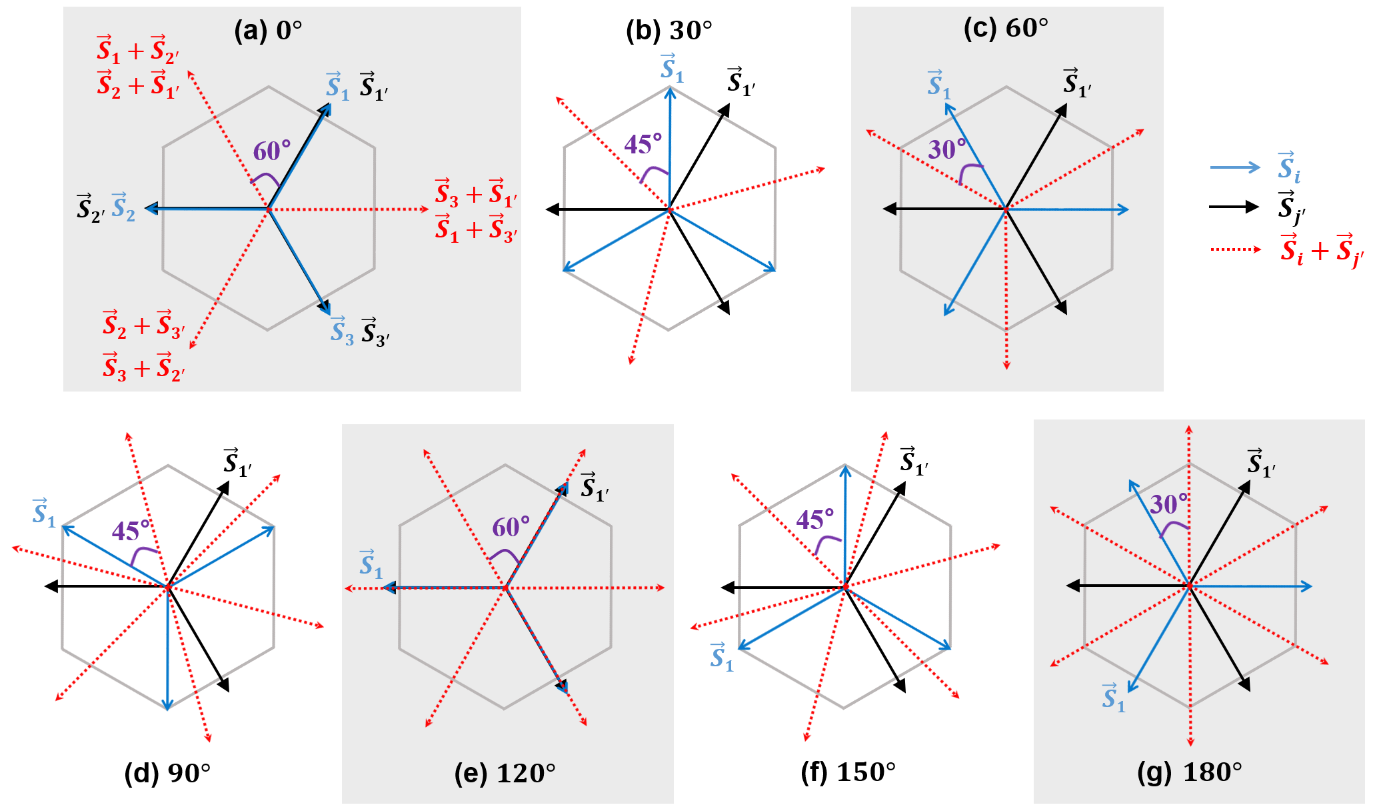


Supplementary Figure 2. The diagrams (a-g) demonstrate the symmetries depending on the spin configuration as the three spins $\boldsymbol{S}_{i}$ in a trimer in the $z=0$ plane are rotated simultaneously by steps of 30 degrees from 0 to 180 degree. Spin configurations (a), (c), (e), and (g) only are commensurate with the triangular symmetry. Other notations are explained in the text.

Note that $\vec{S}_{i}+\vec{S}_{j^{'}}$ is a good quantum quantity of the second term in the Hamiltonian given by the Equation (1), since$(\vec{S}_{i}+\vec{S}_{j^{'}})^{2}=S_{i}{}^{2}+S_{j'}{}^{2}+2\vec{S}_{i}\bullet\vec{S}_{j^{'}}$. Therefore, we would look into the quantity $\vec{S}_{i}+\vec{S}_{j^{'}}$commensurate with the symmetry of the triangular lattice. Suppose that all the three $\vec{S}_{i}$ spins in one trimer are rotated by certain degrees through the resonance of the excitation light on the sample, while neighboring spins remain the same. The notations in Supplementary Figure 2 are the same as in the Equation (1). Blue arrows represent the directions of $\vec{S}_{i}$ which are in one trimer of the Mn^3+^-ion spins in the $z=0$ plane. Black arrows express those of $\vec{S}_{j^{'}}$ in neighboring trimers projected onto the trimer. In the ground state ($\Gamma_{4}$), all the spins $\vec{S}_{i}$ and $\vec{S}_{j^{'}}$ are lying in the *xy* plane maintaining the AFM configuration. The total vector sum, $\sum_{\begin{aligned} i,j=1 \\ (i\neq j) \end{aligned}}^{3} (\vec{S}_{i}+\vec{S}_{j^{'}})$ should retain zero to keep the AFM ordering even after the spin excitation. Red dotted arrows indicate the direction of each term $\vec{S}_{i}+\vec{S}_{j^{'}}$ in the summation $\sum_{\begin{aligned} i,j=1 \\ (i\neq j) \end{aligned}}^{3} (\vec{S}_{i}+\vec{S}_{j^{'}})$.

From Supplementary Figures 2(a) to 2(g), the directions of $\vec{S}_{i}$ in a trimer (blue), those of $\vec{S}_{j^{'}}$ in neighboring trimers (black), and the directions of each term in the summation $\sum_{\begin{aligned} i,j=1 \\ (i\neq j) \end{aligned}}^{3} (\vec{S}_{i}+\vec{S}_{j^{'}})$ (red) are shown as the three spins $\vec{S}_{i}$ in a trimer in the $z=0$ plane are rotated simultaneously by steps of 30 degrees from 0 to 180 degree. It is clear that the spin configurations shown in (a), (c), (e), and (g) are commensurate with the triangular symmetry, while those shown in (b), (d), (f) are not.


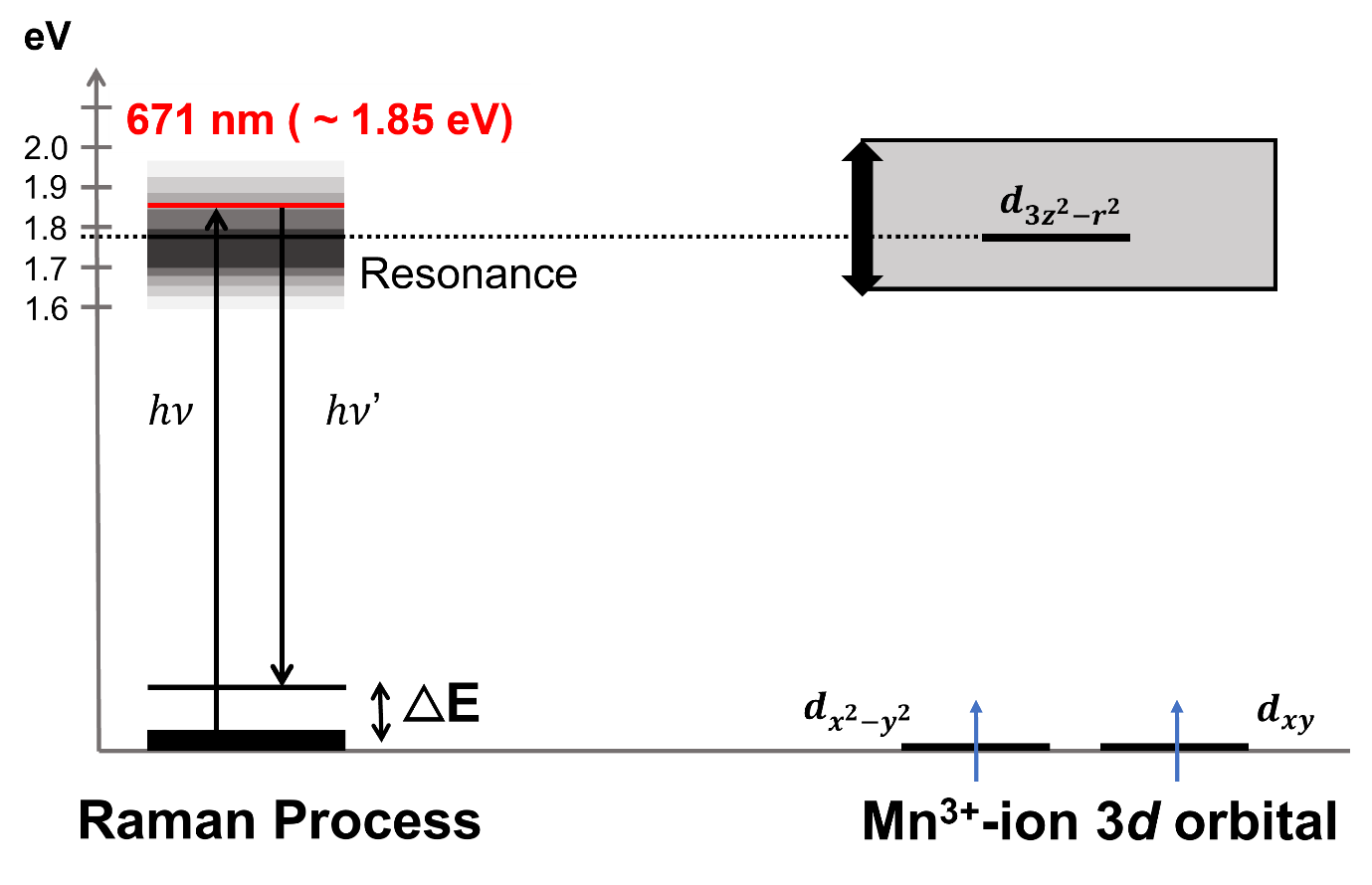


Supplementary Figure 3. Energy diagram of Raman scattering process in MnO_5_ bipyramid of hexagonal LuMnO_3_. Raman scattering associated with Mn *d-d* transition would occur near 1.79 eV by the incident laser. The gray gradation left side depicts the density of electronic states of LuMnO_3_ at 10 K. The energy difference, $\Delta E$, would be corresponding to the spin-rotational excitations described in the manuscript.

Supplementary Figure 3 is displayed to explain the spin excitations measurement by resonance Raman spectroscopy in the hexagonal LuMnO_3_ in details.

Raman scattering in general is one of the inelastic scattering of light from elementary excitations in a material. There are three steps: the virtual absorption of an incident photon of frequency $\nu$, followed by the creation (Stokes Raman) or destruction (anti-Stokes Raman) of an elementary excitations which could be phonon, magnon, superconducting gap, and spin excitation as in this manuscript, with the subsequent emission of a scattered photon $\nu'$. By the energy conservation, we could find the energy of elementary excitation;

$\Delta E=h\nu-h\nu'$ .

$\Delta E$ is the energy of elementary excitation, $h\nu$ is incident light energy, and $h\nu^{'}$ is scattered light energy. The usual energy scale is $\left| h\nu\right| \approx\left| h\nu^{'} \right| \gg\Delta E$, and Mn *d-d* transition (~1.7 eV) has resonance with $h\nu$ or $h\nu^{'}$ in our case. On the other hand, $\Delta E$ is attributed to the excitation energy of the spin rotations in the order of 20 ~ 100 meV. The absorption of the incident light and the emission of the scattered light take place much shorter in time scale than the lifetime of the elementary excitation, spin-rotational excitation in this case.
